# Tissue tectonics and the multi-scale regulation of developmental timing

**DOI:** 10.1098/rsfs.2020.0057

**Published:** 2021-04-16

**Authors:** Lara Busby, Benjamin Steventon

**Affiliations:** Department of Genetics, University of Cambridge, Cambridge CB2 3EH, UK

**Keywords:** developmental, timings, review, tissue, tectonics, multi-scale

## Abstract

Development encompasses processes that occur at multiple length scales, including gene-regulatory interactions, cell movements and reorganization, cell signalling and growth. It is essential that the timing of events in all of these different processes is coordinated to generate well-patterned tissues and organs. However, how the timing of intrinsic cell state changes is coordinated with events occurring at the multi-tissue and whole-organism level is unknown. Here, we argue that an important mechanism that accounts for the integration of timing across levels of organization is provided by *tissue tectonics*, i.e. how morphogenetic events driving tissue shape changes result in the relative displacement of signalling and responding tissues and coordinate developmental timing across scales. In doing so, tissue tectonics provides a mechanism by which the cell specification events intrinsic to cells can be modulated by the temporal exposure to extracellular signals. This exposure is in turn regulated by higher-order properties of the embryo, such as their physical properties, rates of growth and the combination of dynamic cell behaviours, impacting tissue morphogenesis. Tissue tectonics creates a downward flow of information from higher to lower levels of biological organization, providing an instance of downward causation in development.

## 1. Introduction

Time is central to biological phenomena: all biological processes are inherently dynamic, and this is true across fields. Developmental biology provides a strong context to study biological time, as it allows for the study of developmental timing at many different levels of biological organization—opening the possibility for the identification of mechanisms that coordinate these different length scales. Developmental timing can be thought of in terms of the absolute timing of a given event, the ordering of events relative to one another, the directionality of developmental processes, and the more general tempo (speed) at which development proceeds [1–3].

We will focus specifically in this review on how the *absolute timing* of a given event in development is controlled and propose a mechanism by which timing may be coordinated across different levels of organization in the embryo. Our aim here is to take a multi-scalar perspective on the analysis of developmental timing, emphasizing how timing at one level of a biological system (for example, the single-cell level) can be modulated by events happening at the cell population, tissue and multi-tissue levels. In doing so, we will highlight how timing is a highly distributed phenomenon in development that can be discussed in terms of emergent properties—how processes can be observed to occur at a higher organizational level from dynamic processes occurring at lower levels. We will also highlight how the reverse can be true, such that the dynamics of lower-level processes are regulated by alterations at higher levels, through downward causation. While this viewpoint abdicates the search for a single causative source of a developmental timer, we believe this holistic view to be essential for the development of meaningful explanations for core developmental principles such as self-organization, pattern regulation and developmental robustness to heterochronic shifts in evolution.

### 1.1. Intrinsic and extrinsic timers

**Figure 1.**
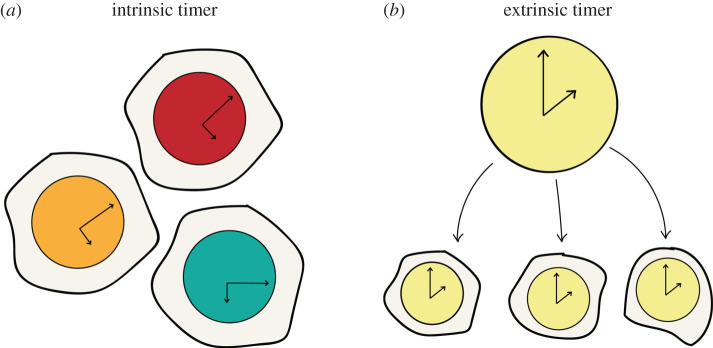
Intrinsic and extrinsic timers. Schematic summarizing the distinction between intrinsic and extrinsic timers. Each of the ‘blobs’ represents either a cell population or cell, dependent upon context. In an intrinsic timer mechanism, each population (cell) has its own internal timer, which is not affected by external information. By contrast, in an extrinsic timer mechanism, information from the surroundings is integrated by the population (cell) to infer the state of the timer.

As a cell moves through developmental time, it undergoes a series of cell state transitions that ultimately define its fate. In considering the mechanisms that regulate the timing of cell state transitions, a distinction has been made between intrinsic and extrinsic timing mechanisms. *Intrinsic* timers function within the cell, while *extrinsic* mechanisms implicate the importance of the external cellular environment in providing inputs to the timer (figure 1). Whether a timer is controlled through intrinsic or extrinsic mechanisms has primarily been investigated using classical experimental embryological methods. For example, physically grafting cell populations between embryos of different ages (heterochronic grafting) allows any influence of external factors on a timer to be identified. If the timer of interest progresses as expected from the age of the donor tissue once placed in this novel environment, it suggests that the functioning of the timer is intrinsic to the cell population. Conversely, if the timer is accelerated, decelerated, reset or advanced in the host context, it suggests that external factors to which the cell population is exposed in this context are important for the normal functioning of the timer and that the mechanistic basis for the functioning of the timer is extrinsic.

To determine the contribution of extrinsic or intrinsic components to timing an event, an additional experimental embryological approach involves removing groups of cells from the embryonic environment and culturing them in a neutral environment (explant culture). If the timer is able to progress outside of the embryo, this suggests that its underlying mechanisms act cell-intrinsically. Note that these experiments are very similar to those used to investigate cell specification and determination. A cell may be defined as specified to form a particular structure if, when isolated from the embryo and placed in a neutral environment, it will still form that structure in the absence of any external inputs [4]. A cell is determined when it gives rise to this structure in any context, including any embryonic context [4]. This differs somewhat from the employment of these assays in the field of timing, but the assays are nonetheless fundamentally the same and highlight the importance of understanding the mechanisms by which intrinsic timers are modulated by the local signalling environment that they encounter during development.

### 1.2. Signalling and intrinsic timers

The specification of distinct cell types during development is inherently linked to the timing at which cells receive either the inhibition or activation of extracellular signals. One example of this is in the patterning of the early ectoderm into epidermis, neural plate border or neural cell states, and the subsequent patterning of these embryonic territories. Neural specification requires a continued modulation of FGF, Wnt and BMP pathway activity from pre-gastrulation stages onwards [5–8], and neural plate border specification and regionalization require a distinct series of temporal exposure to these same pathways [9–13]. Hence, an important unanswered question in developmental biology is how the temporal exposure to extracellular signals is regulated during early development, and how this is linked to alterations in the morphogenetic properties of tissues as they undergo shape change and growth. This understanding is essential, as it likely holds the key to understanding the regulative and self-organizing properties of the early embryo.

**Figure 2.**
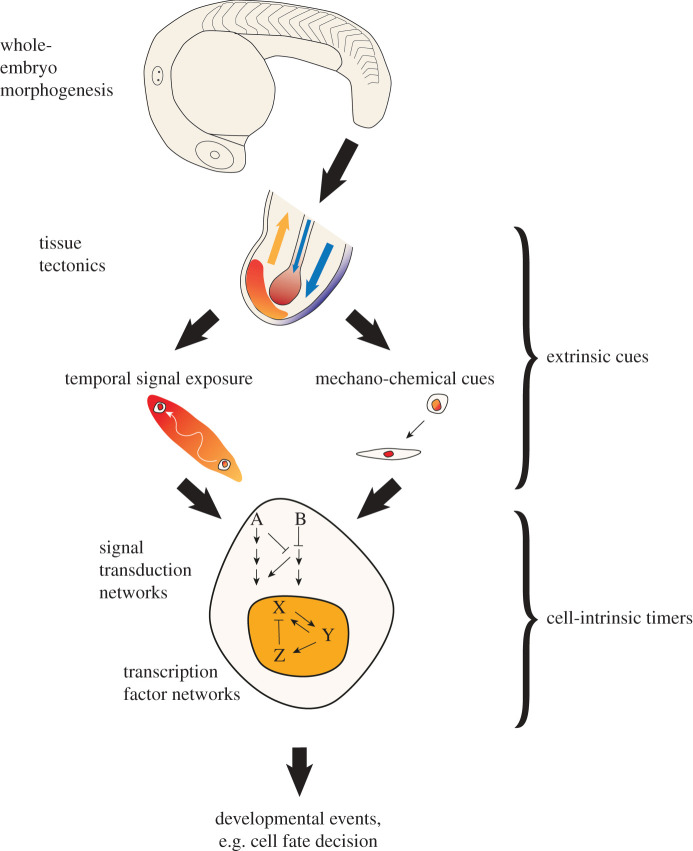
Mechanisms of downward causation in development. This schematic shows the different ways in which morphogenesis can influence cell-intrinsic transcription factor networks and ultimately the timing of developmental events. This can occur both via mechano-chemical cues (as described elsewhere—see text for references), and, as we propose here, through the modulation of temporal signal exposure. As whole-embryo morphogenesis occurs (top panel), signalling and responding tissues move relative to one another (tissue tectonics, second panel). Thus, the exposure of the responding tissue to signals changes over time. Signals feed into cell-intrinsic signal transduction networks that ultimately converge on transcription factor networks. In this way, extrinsic cues interface with cell-intrinsic timing mechanisms via tissue tectonics.

The timing at which cells receive external signals to modulate intrinsic timers of cell state transitions is in turn determined by when the two cell populations (i.e. signalling and responding populations) become opposed to one another in the embryo, or become shifted relative to the position of cells releasing secreted modulators of the signalling pathway activity (figure 2, left branch). Therefore, a key regulator of developmental timing acts at the multi-tissue level and is based on the progressive spatial repositioning of tissues as they alter in size and shape through morphogenesis. To emphasize the importance of this higher-level regulation of developmental timing through the spatial displacement of signalling and responding tissues, we re-introduce the term *tissue tectonics*. This term has been introduced elsewhere in relation to the tension and stress forces acting within tissues to drive morphogenesis [14]. Here, we extend the concept to consider how it can act as an important regulator of timing in development and provides a mechanism by which intrinsic developmental timers can be regulated by morphogenetic events. In addition to altering the temporal exposure to extracellular signals, tissue tectonics also likely impact intrinsic timing via direct mechanical impact on cells (figure 2, right branch). While the direct regulation of cellular signalling and gene expression states by mechano-chemical coupling has received increasing attention [15,16], this review highlights tissue tectonics as a mechanism by which the temporal exposure to signals is linked to multi-tissue morphogenesis and patterning in development. We propose that changes to tissue tectonics in evolution, and more generally alterations to the timing of developmental events (heterochrony), are important for producing new forms.

As a complete coverage of the literature on developmental timing would be beyond the scope of a single review, we instead provide a series of case studies in which the concept of developmental timing has been approached at different levels of biological organization. We will first present a series of studies that have described intrinsic cell timers, including those that contribute to species-specific differences in timing (tempo). We will then use two case studies to show how these intrinsic timers can be modulated at specific windows in development through the movement of tissues during embryogenesis. We will then briefly discuss how alterations in the timing of extrinsic signal exposure can act as a mechanism for evolutionary change in reference to a fundamental concept in evolutionary developmental biology: heterochrony. Together, these examples implicate the importance of signalling events between tissues as points of control in developmental timing, supporting our central thesis that tissue tectonics is important in coordinating developmental timing across levels of organization. Finally, we will review some recent work that demonstrates the importance of tissue tectonics in coordinating multi-tissue morphogenetic events with the timing of cell fate decisions and the emergence of spatial patterning in development.

## 2. Intrinsic and extrinsic timers in development

### 2.1. Cell-intrinsic timers

As a starting point to consider how developmental timing is regulated during embryonic development, we will first consider examples from the literature where the intrinsic capability of individual cells has been demonstrated experimentally. The concept of cell-intrinsic timers is inherently linked to the concept of competence in development: how cells change in their ability to respond to a given inductive cue over developmental time. Induction may be defined as the process in which an inducing tissue releases a signal that results in a change in the direction of differentiation of a responding tissue [17]. In *Xenopus* animal cap explants, there is a clear delineation in time in the transition between a competent and a non-competent state for mesoderm induction: the competence of animal cap tissue to respond to contact with vegetal tissue is lost at the early gastrula stage [18]. To investigate what determines the timing of competence in development, experiments have been performed that isolated animal cap ectoderm from the embryo and showed that, even outside of the embryonic environment, competence is still lost at the same point in time [19]. These results indicate that competence loss is intrinsically timed. Further, even when cells are dissociated from the animal cap, single cells maintain the expected timing of competence: this suggests that timing acts in this case cell-autonomously [19]. By placing dissociated single cells in a solid gelatin matrix, the authors inhibited cell division and showed that loss of competence in this context is independent of cell division [19]. Together, these results implicate a cell-intrinsic timer in animal cap ectoderm cells that modulates the ability of the cell to respond to induction by vegetal signals.

An additional example of single-cell-intrinsically timed developmental events comes from the *Drosophil*a nervous system. Here, neuroblasts give rise to the precursors of the nervous system, ganglion mother cells (GMCs). GMCs are produced by asymmetric divisions of a neuroblast that produce a daughter neuroblast and a daughter GMC. Through the sequential expression by the neuroblast of ‘temporal identity genes' (*hunchback, kruppel, pdm1, castor*), each of the sequentially generated GMCs has a specific identity [20]. How are the transitions between each of these gene expression profiles controlled over time? Experiments that cultured isolated neuroblasts *in vitro* showed that gene expression transitions occur in isolated cells outside of the embryonic environment, suggesting that this gene expression timer is controlled by cell-intrinsic mechanisms [21]. In G2-arrested embryos, the timing and order of expression of the temporal identity genes are maintained, suggesting that the timing of these transitions is regulated by a mechanism independent of the cell-cycle [21]. This is also not a simple linear positive transcriptional cascade; mutations in the *hb* and *Kr* genes have little effect on later gene expression of the other temporal identity genes. More recently, mathematical modelling work has provided insights into the mechanistic basis for the *Drosophila* neuroblast temporal identity timer. Experimental data are consistent with a repressor-decay timer, where the decay of a previous timer component (e.g. *hb*) times the onset of expression of a later component (e.g. *pdm1*) through the relief of repressive interactions [22].

An additional example of an intrinsic timer shown to function in individual cells is given by the oscillatory component of the segmentation clock. During the production of the embryonic anteroposterior (head to tail) body axis, paraxial mesoderm on either side of the midline is sequentially segmented into blocks termed somites (somitogenesis). The primary model for somitogenesis is the clock and wavefront model, in which an anteroposterior gradient of FGF and Wnt signalling (the wavefront) is combined with a cell-intrinsic oscillator based on Notch signalling to segment blocks of tissue [23,24]. A set of genes including those that encode transcription factors of the Hes/Her family are expressed with oscillatory dynamics in the presomitic mesoderm (PSM) [25,26]. Strikingly, when tailbud cells are removed from zebrafish embryos and cultured *in vitro* with recombinant FGF, oscillations in Her1 expression are still observed [27]. Mathematical modelling was used to demonstrate that the observed patterns of gene expression are consistent with all of the cells essentially having the same oscillatory behaviour captured at different points in their dynamics [27]. Thus, in development, individual cells have the capacity to generate oscillatory gene expression, which must be coordinated across the PSM population through extrinsic signals (recently reviewed in [28]). This cell-intrinsic oscillator is important, together with extrinsic signals (the wavefront), in segmenting blocks of paraxial mesoderm along the anteroposterior axis.

#### 2.1.1. Species-specific developmental tempo

An additional example of cell-intrinsic timing mechanisms comes from a key set of studies that have recently investigated the developmental basis for species-specific differences in developmental timing. The pace (tempo) and the duration of embryonic development are highly variable among different animal species. For example, even among mammals, variation in the gestation period is vast. Mouse embryonic development typically spans 20–30 days (dependent on the species), while human development takes nine months. Many of the developmental processes encompassed within embryogenesis differ in their pace between species, and so an active field of research focuses on asking how different tempos of development are achieved in different organisms. A study that differentiated mouse EpiS cells and human ES cells to neural fates in culture demonstrated that species-specific developmental timing is maintained in culture, with neural differentiation markers expressed with accelerated timing in the mouse cells [29].

A recent study used mouse and human cells in culture to investigate the basis for species-specific differences in the tempo of the segmentation clock [30]. Matsuda and colleagues set up a cell culture system to differentiate pluripotent stem cells to a PSM-like state. They used a luciferase reporter gene under the control of the *HES7* promoter in order to image oscillations in gene expression over time, finding that the period of oscillations differs between human and mouse cells (in culture)—the period of oscillations is 2–3 times longer in human cells than in mouse cells. This cell culture assay allowed the authors to ask how different species' cells, cultured in identical conditions, exhibit different oscillatory periods of gene expression. Experiments that swapped the *HES7* loci between cells of each species (inserting the mouse *HES7* locus into the human cell genome, and vice versa) demonstrated that the difference in period is not a result of sequence differences at this locus. Measurement of biochemical reaction parameters revealed differences in the rate of degradation of HES7 protein in human cells relative to mouse cells, regardless of whether this protein was encoded by the mouse or human gene sequence. Further, the delay in transcription and translation of *HES7* was greater in human cells than mouse cells. Using mathematical modelling, the authors showed that altering biochemical reaction parameters in line with these observations is sufficient to account for the different periods of oscillation in human and mouse cells [30]. These results suggest that some cell-intrinsic fundamental difference in the status of biochemical reaction parameters may be responsible for differences in tempo of the segmentation clock.

Similar experiments that differentiated mouse and human spinal cord progenitors to form motor neurons in culture revealed global differences in protein stability between mouse and human cells [31]. Motor neuron differentiation in the embryo takes 3–4 days in the mouse and around 2 weeks in humans [32]. The pace of gene expression progression in progenitor cells in culture closely resembles the differences observed in embryos during this process. For example, the expression of motor neuron marker genes including *Isl1* occurs after 2–3 days of culture in mouse cells but not until approximately 6 days in human cells [31]. These differences do not result from genomic sequence differences, as the introduction of the human *Olig2* gene into mouse cells reveals that the timing of expression of this gene is determined by the cellular context: the human *Olig2* gene in mouse cells is expressed with the same timing as the mouse *Olig2* gene in mouse cells. Given that the observed differences in the timing of motor neuron differentiation do not result from sequence differences in the known gene-regulatory network components, the authors assayed kinetic parameters of gene expression in order to look for differences between mouse and human cells. They found that, while mRNA half-life was similar in the two cell types, protein half-life was significantly shorter in mouse progenitors than in human progenitors. The authors argue that this is a general pattern, because the introduction of an exogenous reporter protein showed that this protein also has a species-specific half-life dependent on its cellular context [31].

These studies investigating the basis for species-specific developmental tempos are intriguing. A species-specific complement of transcription rate, translation rate, mRNA and protein stability may determine the tempo of embryonic development. Interestingly, a study that compared eight different rodent species showed that protein degradation rates are negatively correlated with species lifespan [33]. Importantly, these species differences appear to be cell-intrinsic to the extent that the introduction of an exogenous gene leads to its kinetics resembling that of the endogenous ones. Important open questions remain: What is responsible for these differences in kinetics? Are genomic sequence differences in the machinery of transcription/translation/protein degradation responsible? Could there be inputs from metabolism? Metabolism and nutrition are key factors that vary dramatically between species: many species that have a prolonged period of embryogenesis also access a large nutrient supply. Studies in wild red deer have shown a modest extension of gestation time in mothers who had lower access to food, suggesting input from metabolism and nutritive factors to development [34,35]. Further research effort is required in this area in order to understand the factors that cause differences in biochemical kinetics and ultimately give rise to species-specific tempos.

The numerous examples outlined above provide good evidence for the existence of cell-intrinsic timers, many of which are able to operate in isolated single cells. Furthermore, they provide examples of the utility of experimental assays in investigating the intrinsic or extrinsic control of developmental timers. Together with methods for the improved imaging and analysis of real-time changes in single-cell gene expression, experimental assays such as those described above are key in enabling questions to be asked relating to the degree to which single-cell-intrinsic timers can be coordinated across groups of cells in a given tissue. While we have outlined the extensive evidence for cell-intrinsic timers, a full understanding of timing in development must consider how these can be tuned by extrinsic mechanisms, as cells do not exist in isolation in the embryo.

We will now turn to two specific case studies that demonstrate the ways in which intrinsic timing mechanisms can be modulated by extrinsic signals—moving our discussions from the single-cell level, to the population and multi-tissue levels of organization.

### 2.2. Balancing intrinsic and extrinsic timing mechanisms in avian limb development

Heterochronic grafting has provided good evidence for a number of population-level intrinsic timing mechanisms in limb development. Here, cells of the polarizing region (or zone of polarizing activity, ZPA) express *Sonic Hedgehog* (*Shh*) for a defined duration between Hamburger–Hamilton stages HH20–27 (approximately embryonic days 3.5–5.5) [36]. Sonic Hedgehog (Shh) protein functions in the specification of anteroposterior positional values and the proliferation of limb bud cells [37,38]. If the polarizing region of an HH20 embryonic wing is grafted in place of the endogenous polarizing region in an HH24 embryo, the donor polarizing region continues to express *Shh* 32 h post-graft. At this time point, the host polarizing region in the contralateral wing has downregulated *Shh* expression and transcripts are not detectable by *in situ* hybridization [39]. This result suggests that the mechanisms controlling the timing of *Shh* transcription in this tissue are not dependent upon extrinsic signalling. Population-level analyses of cell-cycle parameters showed that there are distinct stereotyped changes to the progression of the cell-cycle between HH20–30, including a marked increase in the proportion of cells in G1 [39]. Inhibiting cell division in the limb bud using the drug colchicine leads to an extension in the period for which *Shh* is expressed [39]. Together, these experiments point to an intrinsic timer controlling *Shh* expression in the polarizing region, with cell-cycle progression as an important input.

A second cell population intrinsic timer acting in avian limb development controls the termination of limb bud outgrowth [40]. The limb field is initially specified as a portion of the lateral plate mesoderm, before outgrowth to form a bud beginning at HH18. The cells of the limb bud proliferate and drive outgrowth. The processes controlling the termination of outgrowth are important for regulating the size and shape of the limb. The classical model for limb outgrowth termination implicated the breakdown of a feedback loop between FGF signalling from the apical ectodermal ridge (AER) and BMP signalling in the underlying mesenchyme [41]. A recent study has produced evidence that a cell-intrinsic timer is key in controlling limb bud outgrowth termination [40]. When donor HH29 distal mesenchyme cells were grafted into a younger (HH20) host limb, grafted tissue had a cell-cycle profile more similar to HH29 control (ungrafted) tissue than to HH24 contralateral tissue [40]. Furthermore, the expression of genes involved in the *TGFβ* pathway, as well as functional BMP signalling, was maintained in grafted tissue with a transcriptional profile similar to HH29 ungrafted tissue [40]. The authors argue that an intrinsic programme of increasing BMP signalling in distal mesenchyme is responsible for the termination of limb bud outgrowth.

An important example of an intrinsically controlled developmental timer is found in the specification of avian limb proximodistal values. The proximodistal axis of the limb comprises three segments: the stylopod, zeugopod and autopod (from proximal to distal). The contribution of progenitor cells to more distal segments of the wing (zeugopod and autopod) is controlled by an intrinsic timer mechanism [42]. Evidence for this intrinsic timer comes from the expression of the autopod marker, *Hoxa13*, in HH20 to HH24 heterochronic grafts. Donor tissue in this context expresses *Hoxa13* with an expression pattern distinct from surrounding tissue, suggesting its fate is not reset upon grafting to this new context. This timer mechanism is associated with an intrinsically timed change in cell adhesion properties. Interestingly, in this example, the intrinsic timer is understood to function from HH20 onwards. Prior to this point, flank-derived retinoic acid (RA) causes cells to take stylopod (proximal) fates. As the limb bud grows and progenitor cells are displaced from the source of RA, they are released from the influence of this extrinsic timer and there is a switch to intrinsic control of cell fate. In this way, an intrinsic timer can be modulated by an RA-based extrinsic timer. This is an example of the *tissue tectonics* concept that we propose here—in this case, physical tissue movements of the limb progenitors away from the flank allow extrinsic modulation of an intrinsic timer mechanism. Thus, a link between cell-intrinsic timing and extrinsic cues is provided by *tissue tectonics*—the relative displacement of signalling and responding tissues.

Taken together, these examples from avian limb development clearly demonstrate the utility of experimental embryological techniques in investigating the contribution of cell-intrinsic and cell-extrinsic timers to the regulation of developmental events. Importantly, studies of proximodistal patterning in this system provide an example of the interplay between intrinsic and extrinsic timers through *tissue tectonics*. Here, the functioning of the intrinsic timer is carefully modulated through physical tissue movements, altering the exposure of cells to an extrinsic signal.

We will now turn to another developmental system where there is good evidence for intrinsic timers that are also likely modulated by extrinsic signals: mammalian neurogenesis.

### 2.3. Balancing intrinsic and extrinsic timing in mammalian neurogenesis

The mammalian brain is complex and comprises a large repertoire of diverse cell types. In many cases, neural progenitor cells undergo temporal changes in the types of daughter cells that they produce, allowing for the production of a diverse set of daughter cells over time. For example, progenitor cells of the mouse cortex may be maintained in a culture system, where they express molecular markers with similar timing to that observed in the embryo [43]. As differential daughter cell identities are observed over time in the absence of any external signals, this suggests that the transcriptional progressions observed in normal development are mediated by an intrinsic timer. These cultured cells are functional: when grafted back into the embryo, their repertoire of projections closely resembles those found in normal development [43]. These results are similar to those in the *Drosophila* neuroblasts described above, revealing an important role in diverse species for cell-intrinsic timers in the generation of the nervous system.

The timing of differentiation of cells that make up the nervous system may also be determined through cell-intrinsic mechanisms. Experiments that removed rat optic nerve cells from the embryonic environment and cultured them in platelet-derived growth factor (PDGF) demonstrated that oligodendrocyte precursor cells (OPCs) divide and differentiate with timing that closely replicates that observed within the embryo [44]. If single OPCs are plated in individual microwells, they divide (with a maximum of eight divisions observed) before differentiating. If separated into individual wells, the daughters of the same OPC divide the same number of times before differentiating [45]. What determines the timing of differentiation in these cells? It appears that the underlying mechanism is cell-intrinsic, given that cells, even in isolation, differentiate with replicable timing. In contrast with the aforementioned examples from limb development, however, this mechanism does not appear to be cell-cycle-dependent. When OPC cultures are held at a reduced temperature (33°C as opposed to the standard 37°C), they divide more slowly. However, the timing of differentiation occurs earlier in these cultures [46]. This suggests that it is not the number of cell divisions that is important, so much as the absolute passage of time. Further investigation has led to a model for OPC differentiation that places gradual changes in the level of several proteins, including p27, p18 and Id4, at the centre of the intrinsic timer (reviewed by [47]).

Gradual changes in transcription over time have been implicated in another study focusing on mammalian cortex development. Here, apical progenitor cells (APCs), which give rise to cells of the dorsal cortex, were subjected to transcriptional analysis at several time points in development [48]. Through computational manipulation of the resulting dataset, the authors were able to distinguish two orthogonal axes in the clustered dataset—a temporal axis and a differentiation axis. That is, a subset of genes was identified whose expression changed over time in a manner distinct from the phenomenon of differentiation. These genes show gradual changes in expression over developmental time [48]. Manipulations that overexpressed a Cyclin-dependent kinase (Cdk) inhibitor through electroporation did not disturb the temporal change in the temporal axis genes [48]. This is evidence for another cell-intrinsic timer in mammalian cortex development that is not dependent upon cell-cycle progression for its function. These results, together with those of the studies described above, implicate an important role for cell-intrinsic timers in generating the highly diverse cell type repertoire of mammalian brains.

The numerous studies outlined above provide strong evidence for the importance of intrinsic timing mechanisms within cells, both in modulating the expression of cell fate markers in developing neural tissues and in the timing of cellular differentiation. As we previously discussed in the context of avian limb development, cells do not exist in isolation in the embryo. The studies described here culture progenitor cells *in vitro*. In normal development, these intrinsic timers are likely modulated by extrinsic signals. For example, what determines when the intrinsic timer ‘starts’ its sequence of events? Given the regulative nature of mammalian development, it is highly improbable that an intrinsic timer functions from the beginning of development.

Heterochronic grafting has provided evidence for population-level extrinsically controlled timer mechanisms in neural development. Progenitor cells of the mammalian neocortex produce different daughter cell fates sequentially during development. Radioactive labelling of progenitor cells in ferrets has shown that the deepest layers of the cerebral cortex are generated before the more superficial ones [49–52]. In experiments that grafted dissociated cells from E29 embryos (which would normally contribute to layer 6 (L6) of the cortex) to embryos at a later stage (in the process of generating L2/3), it was found that, dependent upon the cell-cycle status of the donor cells, a different outcome was observed [53]. If donor cells were transplanted into the older host embryo prior to cell-cycle completion (during S-phase), daughter cells contributed to L2/3 of the cortex, like host progenitor cells at this stage. However, if cells were allowed to complete their cell-cycle before transplantation (the graft was performed 24 h later), daughter cells were found in L6 [53]. These experiments suggest that there are extrinsic inputs to the timer mechanism that controls the progressive generation of different neuronal fates by these progenitor cells in this context. It remains to be seen what these signals are and why.

Studies in mammalian neurogenesis provide evidence for the importance of balancing intrinsic and extrinsic timing inputs in development. A number of studies have shown the existence of intrinsic timers in this system, implicated in the control of transitions between generating different neural cell types as well as the timing of differentiation of individual progenitor cells. In this example, it remains to be seen what the extrinsic cues that feed into cell-intrinsic systems are, and how they are modulated in time and space by the movement of tissues relative to one another.

We will now turn to a consideration of evolutionary changes to the timing of signalling events at the multi-tissue level—here, changes to tissue tectonics result in altered signalling timing (heterochrony) and large-scale morphological changes.

## 3. Time in the evolution of development: heterochrony

Heterochrony, defined as a shift in the developmental timing of events, has long been recognized as an important concept in the field of evolutionary developmental biology (evo-devo). Both De Beer and Gould published influential texts arguing that changes in developmental timing of events relative to an ancestral form are an important force in the evolution of morphology [54,55]. Dramatic changes to morphology may be achieved through changes in the timing of the development of specific organs relative to one another, as demonstrated by the following examples. Here, we will focus on two examples of developmental heterochrony at the multi-tissue level of organization.

**Figure 3.**
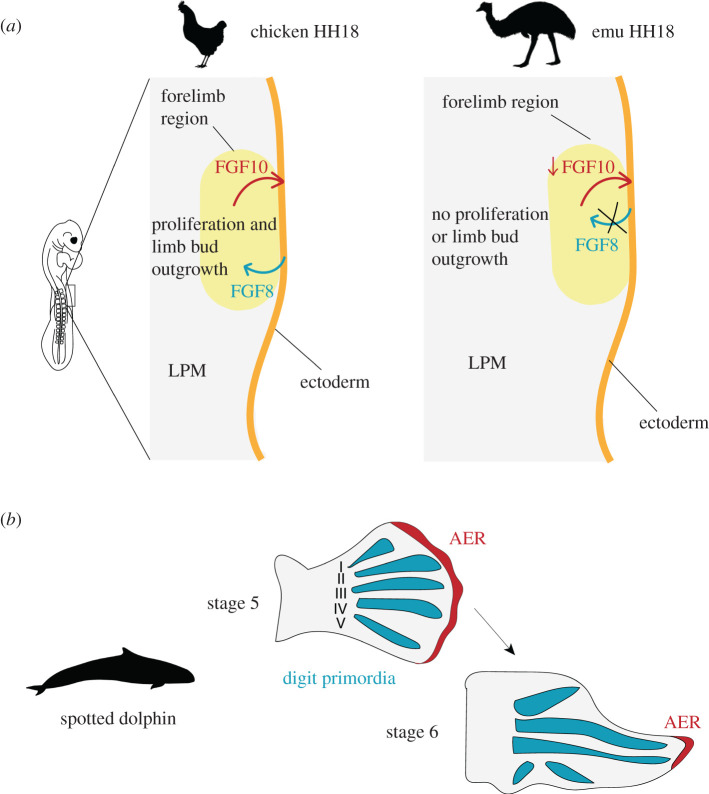
Heterochrony between tissues can produce new forms in evolution. (*a*) Schematic of the chicken and emu lateral plate mesoderm (LPM) at HH18. In most avian species, including the chicken, reciprocal signalling between the forelimb region of the LPM and the overlying ectoderm (orange) promotes proliferation of LPM cells and limb bud outgrowth. However, the emu forelimb region of the LPM produces the signalling ligand FGF10 at an insufficient level to induce FGF8 production in the ectoderm (orange). Consequently, cells of the LPM forelimb region do not proliferate and the limb bud does not grow out from the body at HH17. (*b*) Analysis of spotted dolphin developing limb buds has shown that the apical ectodermal ridge (AER) persists over digits II and III for longer than over the other digits. These digits exhibit hyperphalangy (many finger bones) and it has been suggested that the local persistence of the AER may be related to this trait. Silhouette images of animals were taken from PhyloPic: please see Acknowledgements for attributions.

An example where developmental heterochrony has been implicated in morphological novelty is given by the emu, *Dromaius novaehollandiae*. The emu is one of many species of flightless bird within the ratites (a group within the Palaeognathae) and has very small wings that have a single digit. The timing of forelimb development in the emu is significantly delayed relative to that of other birds and other amniotes. The limb bud does not grow out from the flank of the emu embryo until HH20—while in the vast majority of birds outgrowth begins at HH17 [56]. A recent study has revealed the underlying developmental basis for the delayed outgrowth of the emu wing bud [57]. Prior to limb bud outgrowth, limb precursor cells reside in the lateral plate mesoderm (LPM). In both the emu and the chicken, the epithelial to mesenchymal transition (EMT) of mesenchymal precursors from the somatopleure and their movement to the LPM is intact [57]. However, in the forelimb field of the emu LPM, these precursors do not promote outgrowth of the limb bud at HH17. Analyses of proliferation in chicken and emu fore- and hindlimb regions of the LPM show that, at stages at which chicken fore- and hindlimb regions and emu hindlimb regions are proliferating, the cells of the emu forelimb LPM do not proliferate [57]. This difference results from a disruption of reciprocal signalling between the LPM and overlying ectoderm. In chicken development, the production of FGF10 by the LPM induces reciprocal signalling via FGF8 from the ectoderm to the LPM [58]. FGF8 signalling of the ectoderm to the LPM is required to promote proliferation in the limb bud field [58]. However, in the emu, the ectoderm overlying the LPM does not express *Fgf8* at HH18 [57]. Grafting of donor chicken LPM into the emu limb field (under the host ectoderm) results in the development of a precocious limb bud, revealing that the difference in limb bud outgrowth timing results from changes to the LPM in the emu [57]. Though *Fgf10* is expressed by the emu LPM in the forelimb region, the authors suggest that the quantitative level of expression of this signalling ligand is insufficient to induce the expression of *Fgf8* in the ectoderm (figure 3*a*). In support of this hypothesis, the overexpression of *Fgf10* in the emu LPM results in *Fgf8* induction in the overlying ectoderm and precocious limb bud outgrowth [57]. An enhancer mutation responsible for the observed differences between *Fgf10* expression in the chicken and emu embryo was also identified.

Together, these results reveal that subtle changes to the timing of expression of a signalling ligand in development are able to alter the timing of development of the emu forelimb, contributing to changes to the gross morphology of this structure. This example is consistent with a role for tissue tectonics in the control of developmental timing, as it implicates the importance of the signalling event in control of downstream events. In this case, the signalling and responding tissues remain apposed but the loss of the signalling ligand expression abolishes the signalling event.

An additional example of heterochrony in morphological evolution is provided by the dolphin flipper. Dolphins are aquatic mammals possessing many adaptations for life in water, including the modification of the forelimb to form a flipper. The flippers act during swimming as rudders, and in many species the digits exhibit hyperphalangy: relative to the ancestral state, they possess numerous finger bones (phalanges) [59]. Careful study of spotted dolphin (*Stenella frontalis*) embryos over the period of limb development revealed that hyperphalangy is localized to digits II and III of the dolphin forelimb [60]. This character correlates closely with the prolonged maintenance of an AER over digits II and III, suggesting that changes to the dynamics of AER development may be responsible for this morphological change [60] (figure 3*b*). Thus, localized persistence of the AER over digits II and III may promote the formation of additional phalanges in these digits.

In summary, it is clear that alterations in phylogeny to the timing of development of specific organs relative to the rest of the body can allow for pronounced changes in form. This pattern was recognized by De Beer [55] and Gould [54], and the examples discussed demonstrate that organ-level heterochrony may occur in diverse contexts. The examples in this section also provide good support for the importance of multi-tissue inductive interactions in the timing of developmental events. In each of these cases (the emu forelimb and the spotted dolphin flipper), changes in signalling tissue dynamics cause a change in the timing of a developmental event. For example, in the emu forelimb, reduced FGF signalling from the LPM to the ectoderm results in a substantial delay in time of a signalling event, and the outgrowth of the limb bud. It is clear that the apposition of signalling and responding tissues (tissue tectonics), as well as underlying signalling dynamics, are important points of control in the timing of events. These signalling events can have effects that span levels of organization: transcriptional changes in receiving tissues, population-level changes (e.g. the outgrowth of limb buds) and organ-level changes (e.g. the timing of development and final morphology of digits of the limb).

## 4. Tissue tectonics as a mechanism to coordinate developmental timing across scales

### 4.1. Pattern emergence in development: how intrinsic and extrinsic timing act together to generate spatial patterns of gene expression

In the experiments enumerated above, we have seen multiple examples where cells display an intrinsic ability to move through successive gene expression states in the absence of extrinsic signals or cues. This demonstrates that cells are not passive entities that await exposure to extracellular signals, but set their own developmental pace of differentiation through a combination of mechanisms. Such mechanisms include the metabolic rate of the cell and the associated tempo of a cell's mRNA and protein turnover and also alterations in the accessibility of transcription factors to bind and regulate gene expression at the chromatin level. Ultimately, these biochemical alterations in a cell's physiology and nuclear architecture will impact the rates of transcription factor production and degradation, as well as the efficiency to regulate either activation or repression of target genes. The impact of these parameters can be modelled together with the higher-level regulative structure of gene-regulatory networks, to generate predictions on the dynamics of cell state transitions through the use of sets of ordinary differential equations [61,62]. When used to simulate the temporal changes in gene expression across a field of cells, this dynamical systems approach has been highly effective in determining how transcription factor networks operate as a function of these dynamic modulators, to give rise to changes in gene expression states over time. Two well-studied examples of how dynamical systems approaches have been used to investigate the function of gene-regulatory networks are the gap gene system in dipteran insects, and the dorsal–ventral patterning of the vertebrate neural tube [63–66]. Locally, autocrine and paracrine signals pass between cells undergoing cell state transitions, allowing for non-cell autonomous regulation of gene expression dynamics across cell populations. When viewed at the tissue level through a series of snapshots of gene expression analyses, coupling cell-intrinsic and cell-extrinsic gene expression regulation in such a way results in the formation of gene expression patterns that are highly striking to the experimental observer. However, it is essential to remember that these gene expression patterns are not established at any one point in time, or through an instantaneous response of gene regulation to external signals. Rather, they are an emergent property of cell-intrinsic regulatory interactions coupled to cell-extrinsic control of their inherent dynamics.

Broadly speaking, extrinsic timers offer a mechanism to provide multi-tissue and multi-organ coordination of developmental processes. Extrinsic signals are inherently linked to the concept of induction in development, and ultimately to the role that morphogens play in patterning tissues as they develop. Historically, the study of morphogens has focused on their ability to generate gene expression patterns at a given fixed point in time and ignores the dynamics of their exposure to cells and that of the emerging gene expression pattern in space. While much can be learned from asking how morphogens can provide sufficient precision in the spatial domain to generate a given pattern, the eventual fate of a cell is not determined by its expression state at any given point in time but is rather an output of the sum of all state transitions it undergoes during development [67]. Caution against viewing pattern formation as a mere ‘snapshot’ of a continuous developmental process has been conceptualized as part of the ‘general relativistic positional information framework’ [68]. This framework places emphasis on our understanding of how biological systems may generate the full dynamic profile of a given set of gene expression states within a tissue of interest, and highlights cells as dynamic entities that integrate multiple sources of information through time (rather than receiving positional information at one critical time point). This consideration re-focuses the question of how gene expression patterns are established away from the generation and interpretation of concentration gradients and towards the regulation of the temporal exposure to morphogens during development.

### 4.2. Tissue tectonics as a higher-order regulator of morphogen exposure during multi-tissue morphogenesis

**Figure 4.**
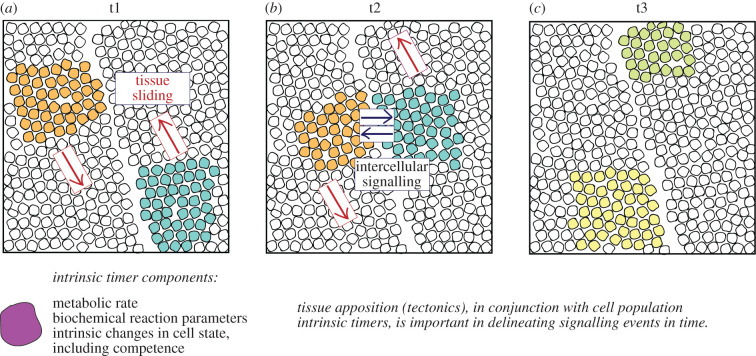
Tissue tectonics. Three snapshots in time are shown in this schematic, at time points t1, t2 and t3. Two populations of cells are shown in orange and teal, each of which is found in different tissue sheets. As the tissues slide relative to one another, the populations of interest come into close apposition, allowing intercellular signalling to occur. At t3, the inductive signalling event has occurred and both populations' cells have experienced a change in cell state. Clearly, tissue tectonics is an important contributor to the timing of signalling events in development.

The timing at which cells and cell populations receive signals is again a highly distributed phenomenon. Signal timing depends not only on when a signal reaches a cell (which itself is a composite of multiple mechanisms of both extracellular and intracellular transport, reviewed by [69,70]), but also on the rate of production and transport of signal inhibitors. Across longer time scales, cells will also move relative to the sources of these signals and inhibitors, creating a patterning mechanism that is acting at the multi-tissue level. The relative rate of tissue movement is an output not only of the state of the cells in question, but also of the mechanical properties of the environment in which it is moving. Here, we term the relative displacement of signalling and responding tissues *tissue tectonics* in reference to the relative sliding of the Earth's plates in the lithosphere (figure 4). The rate at which these multi-tissue interactions occur is inherently linked to alterations in the mechanical properties of tissues as they are formed during development.

To fully illustrate how alterations in tissue tectonics can impact the spatial and temporal regulation of patterning in development, we will briefly review three examples of ways in which tissue movement and shape changes act as extrinsic signals that feed into cell-intrinsic signal transduction networks.

#### 4.2.1. Vertebrate gastrulation

During gastrulation, multiple tissue interactions act together to both specify and pattern the three principal germ layers along three principal axes of orientation: anterior–posterior, dorsal–ventral and left–right. In addition to establishing these essential coordinate systems of the body plan, a series of cell movements act in a well-orchestrated manner to progressively separate layers of tissues, and to begin the process of embryo elongation along the anterior–posterior axis. A particularly well-studied aspect of patterning during this process is the initial specification of neural tissue with the ectoderm, and its subsequent patterning along the anterior–posterior axis. While signals from the early gastrula stage organizer are important for ‘activating’ the initial anterior character of neural tissue, subsequent ‘transforming signals’ then act to convert this character to more posterior neural tissue as gastrulation proceeds (for a recent review see [71]). During both the initial specification of neural tissue and its subsequent patterning, multiple signals are required to integrate together that result in a precise temporal modulation of FGF, BMP, Wnt and RA signalling pathways [5,6,72]. A conserved element of these interactions is a requirement for the downregulation of BMP signalling during neural plate specification, and a subsequent posteriorization by the Wnt signalling pathway [73]. The temporal exposure of ectodermal cells to these pathways has been shown to be a key component of the patterning mechanism [7,8], highlighting the question of what regulates the temporal exposure of cells to patterning signals during gastrulation.

We propose that tissue tectonics is a key aspect of the temporal regulation of signal exposure, and it is essential for ensuring appropriate coordination between the morphogenetic and patterning aspects of gastrulation. One aspect of this coordination is well studied and requires information to flow from gene-regulatory network activity through to the control of cell movements, tissue morphogenesis and embryo elongation. Critically, however, it also requires information flow in the opposite direction, i.e. cells must be able to determine the state of embryo elongation and tissue morphogenesis to coordinate these processes with cell specification and patterning. A recent study has approached this question using explants of zebrafish embryonic cells that were cultured away from the yolk and yolk syncytial layer [74]. Such aggregates go on to break morphological symmetry and generate multiple germ layers in an organized manner [74–76]. As the explants continue to elongate, progressive bands of the hindbrain marker Krox20 appear concomitantly with the movement of a pole of Wnt/beta-catenin away from a source of BMP4/7 expression at the opposite end, suggesting that the elongation itself may be an important upstream regulator to determine the timing of exposure to both BMP and Wnt signal activity. Indeed, blocking convergence and extension of the explants results in an alteration in the spatial–temporal exposure to these signalling pathways and the specification of the hindbrain [74]. Together, these results provide an initial insight into the role that tissue tectonics plays in providing a causal link between the mechanisms of global embryo elongation and the patterning of the nervous system during gastrulation.

#### 4.2.2. Cavefish eyefield specification and the evolution of gastrulation

Alterations in the morphogenesis of gastrulation are common and require a mechanism for such alterations to impact patterning in a manner that a conserved body plan can be generated at later developmental stages. A recent study examined differences between two different morphs of the characid fish during gastrulation: a wild-type river-dwelling morph and a cave morph. While the overall body plan is broadly similar, cavefish possess a number of morphological differences relative to surface fish, including a complete loss of eyes. This opens the question of how alterations in the morphogenesis aspect of gastrulation might impact the patterning aspect of gastrulation in the adaptation of populations to new ecological environments. An examination of the expression of the homologues of various genes expressed by the organizer in the embryos of characid fish revealed substantial differences in the expression of these genes during gastrulation [77]. For example, *dickkopf1b* (*dkk1b*) is expressed in two distinct populations at 50% epiboly in the river-dwelling morph, but in one continuous domain at the same stage in the cavefish morph. This difference in expression domain is associated with advanced internalization of these cells (which contribute to the anterior prechordal plate) in the cavefish morph relative to the river-dwelling morph. Notably, the expression of *dkk1b* is also downregulated earlier in the cavefish morph than the river-dwelling morph. As a consequence of shifted timing of AP axis formation (heterochrony) in the cave morph, the eyefield that forms within the overlying neurectoderm is reduced in size. Through functional experiments that mimicked the impact of advanced Wnt signalling activation in the eyefield (through treatment with LiCl, because *dkk1b* is an antagonist of Wnt signalling), the authors showed that increasing Wnt signalling in early surface-dwelling embryos results in a reduced eyefield and later a misshapen retina. Together, these results give an example of a developmental signalling event that is altered in timing through changes to the timing of apposition of tissues (here, the anterior neurectoderm and the anterior prechordal plate). In this example, changes to the timing of these events have a marked morphological effect, accounting for the loss of eyes in the cavefish morph. This study opens a set of fascinating questions over the limits of developmental constraint and robustness in the evolution of gastrulation morphogenesis, and the causal role that tissue tectonics might play in linking these two aspects of body plan development and evolution.

#### 4.2.3. Neural crest–placode interactions during cranial neural crest migration

The sensory structures of the vertebrate head form from multiple different cell types that can be traced back to two distinct embryonic cell populations that lie along the neural plate border: the cranial neural crest (CNC) and sensory placodes. As they become specified, and undergo collective morphogenesis, differentiation and migration, there exist multiple points of interaction between these two populations that are important for forming appropriately formed sensory structures [78]. One particular set of interactions requires a combination of inter-tissue signalling and morphogenetic behaviours that represent a third example of tissue tectonics in action during development, as has been studied in *Xenopus* embryos. Neural crest cells emerge from the dorsal neural tube as a series of three migratory streams and begin to migrate collectively towards the ventral region of the embryo. Within the deep layer of the ectoderm, an initially common pre-placodal region (PPR) progressively splits up into distinct placodal thickenings via a series of cell movements [79]. Within the region of the CNC migratory domain, future cells of the epibranchial placodes move to form bands of thickened ectoderm between each migratory CNC stream. Explanting both pre-migratory CNC and PPR and culturing them adjacent to one another revealed a ‘chase and run’ behaviour that can be decomposed into two principal interactions. First, a chemo-attractive cue from the PPR that results in the directional movement of CNC cells towards the PPR explant. Second, a contact-dependent repulsive cue meditated by N-cadherin and the PCP signalling pathway that results in a movement of PPR cells away from the CNC. Such behaviour results in a self-assembled coordinated migration of the two populations that aids the progressive patterning of both CNCs in the ventral migration as distinct streams of cells, and the formation of distinct epibranchial placodes that are important for their differentiation in the epibranchial ganglia [80,81]. Importantly, continued interaction of epibrancial placode and CNC derivatives is required at later stages to generate fully functional cranial ganglia that are well aligned and connected to the central nervous system. These interactions represent a third example of how interactions at the level of multi-tissue morphogenesis can play an important role in directing appropriate cell signalling and differentiation events at the level of single cells and collectives.

### 4.3. Tissue tectonics as a mediator of downward causation in development

Historically, developmental biologists have focused on the emergent properties of development: how processes at a lower level of a complex system (i.e. at the level of a cell and the mechanisms driving its cell state transitions) can impact observable features at higher levels (i.e. the patterned expression of genes across a field of cells when observed at a fixed time point). However, one of the salient properties of developmental systems is their ability to regulate pattern upon the loss or experimental removal of certain parts of the embryo. Some striking examples include, but are in no way limited to, the ability of halved sea urchin embryos to each give rise to a fully formed individual, the ability of the chicken embryo to develop normally after surgical removal of a large portion of the primitive streak, and the formation of monozygotic twins [82,83]. This regulative (or self-organizing) ability of developmental systems fascinates experimental embryologists to this day and has recently come back into focus through observations regarding the ability of embryonic cells to break symmetry and generate patterns when aggregated and cultured as multi-cellular aggregates [84–87].

**Figure 5.**
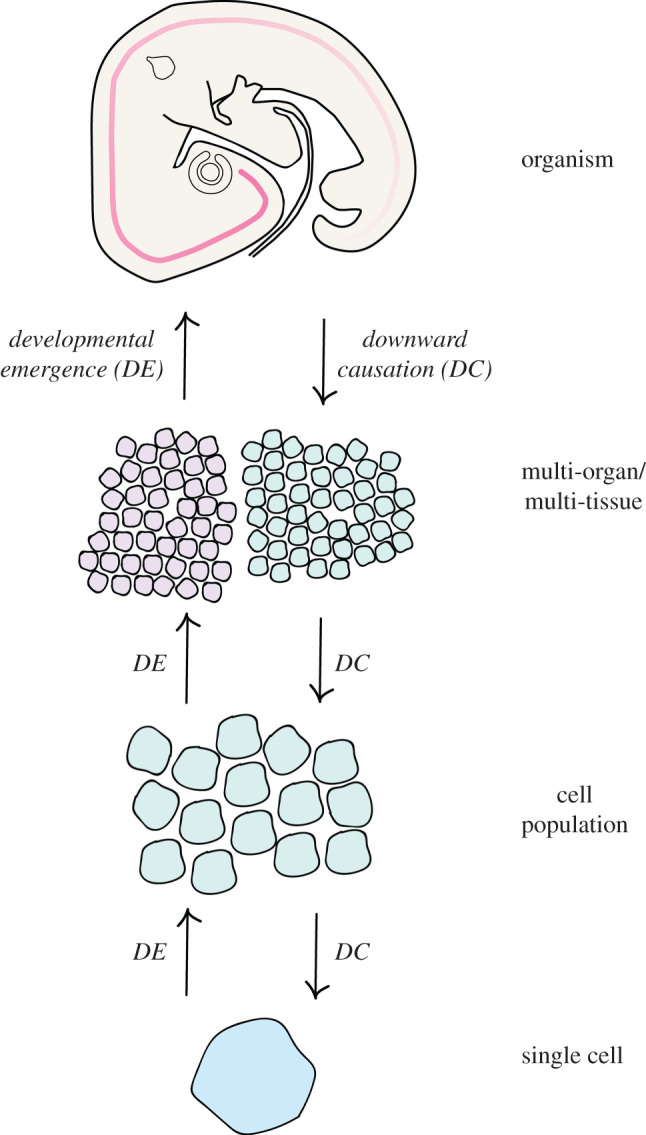
Summary. This schematic summarizes the different levels of organization where timing has been studied in developmental biology. Interaction between the various levels of organization occurs bidirectionally. Information passes from higher to lower levels through *downward causation*, exemplified by pattern regulation in the embryo (see text for examples). Information also passes from the lower levels to higher levels, through *developmental emergence*. For example, changes in cell state can lead to changes to inter-population signalling and ultimately direct higher-level changes to the embryo in morphogenesis. We argue that a link between these levels of organization is provided by the concept of *tissue tectonics*. The ways in which signalling and responding tissues are displaced relative to one another can influence the timing and location of signalling events between tissues.

Regulative development requires a mechanism that enables the sensing of changes to the properties of a system at higher levels (including alterations in the size and/or shape of an embryo or primordium), and to convey changes in the state of the system at lower levels (i.e. alterations in the intrinsic state of cells and cell population) (figure 5). To achieve this, there must be an element of downward causation in the system; essentially, a mechanism by which information can be passed downwards to confer alterations in a cell's gene expression state in response to multi-tissue level perturbation. This downward causation runs in the opposite direction to the emergence of gene expression patterns (figure 5), and similarly requires an understanding of how alterations in the timing of exposure to signals and their inhibitors are regulated through time. We propose that tissue tectonics is also an essential consideration in understanding the mechanisms of downward causation in development, because downward causation is intimately associated with signalling between cells. It is clear to see how manipulations to the embryo (for example, removal of the anterior primitive streak) would impact signalling events between cells, changing the apposition of different tissues. A full understanding of the mechanical properties of tissues will allow us to follow how tissues respond to injury, how this impacts the timing of exposure to extrinsic timers, and how this in turn regulates the operation of intrinsic timers and the emergence of patterns during regulative development and self-organization.

Probing the mechanisms of tissue tectonics in development will require a new experimental framework that requires a return to the approaches of experimental embryology, but with the advantages of new technologies in multi-scalar imaging and targeted tissue manipulations. The era of developmental genetics has provided a ground-up perspective of gene function in development, focusing on the targeted manipulation of gene function to then assess the phenotypic consequences at higher-order levels of the system. In parallel to this, we propose top-down manipulations that perturb morphogenetic events at the multi-tissue level and observe consequences at the sub-cellular, genomic and gene-regulatory levels. Thus, a typical experimental paradigm for probing tissue tectonics would be as follows. First, to gain a quantitative understanding of the morphometric changes to the tissues and cell populations under study. Second, to achieve a spatially and temporally targeted manipulation of these processes, which may be through the use of tissue ablation or through the use of optogenetics to manipulate key regulators of tissue shape change. Third, to observe the impact of these manipulations on the relative positioning of signalling and responding tissues and how this alters the dynamics of cell fate specification. Finally, to investigate the extracellular signalling events that are responsible for providing an extrinsic cue—this may be through the regulation of temporal exposure to signals and signal inhibitors (figure 2; left branch) or through mechano-chemical signalling (figure 2; right branch). While the focus here has been on the study of downward causation from the multi-tissue level, instances will also occur between intervening levels of biological organization, and in these cases the same experimental logic would apply.

### 4.4. Tissue tectonics is not the only way in which alterations in the mechanical environment of tissues and cells can provide a mechanism of downward causation in development

While asking the question of how the timing of exposure to extrinsic signals has led to a focus on tissue tectonics in this review, there is increasing evidence for the role that mechanical forces can play in regulation to gene expression states when applied directly to cells through their changing extracellular environments. When sensing such changes through mechano-chemical signalling pathways, cells thus integrate an additional level of downward causation that has been discussed in detail recently elsewhere [15,16]. There are also recent examples of downward causation in action from the level of multi-cellular rearrangements to the temporal regulation of genetic oscillators acting within cells. One such example is in the regulation of the somitogenesis clock acting within PSM cells and again emphasizes the importance of considering how multiple different time scales can act together during development to generate collective behaviours. As introduced earlier, cells within the PSM operate a cell-intrinsic oscillation of Her/Hes gene expression mediated via a self-repression gene-regulatory module [26,88–92]. Notch signalling is known to locally synchronize these oscillations across a field of cells [93,94]. However, cell rearrangements acting at a different time scale can impact this synchronization by altering the time during which cells can make contact and signal via Delta–Notch interactions [95], and theoretical studies have shown how cell movements observed with the zebrafish PSM progenitor domain are of the right time scale to enhance the synchronization of the somitogenesis clock [96]. Hence, this represents an additional example of downward causation where information flows from the level of cell–cell rearrangements, through cell-to-cell signalling and the synchronization of cell-intrinsic gene-regulatory dynamics [97,98].

## 5. Conclusion

In this review, we have given an overview of studies in developmental biology that have asked how developmental events are timed. These studies have focused on a variety of levels of organization: from the single cell to the coordination of multi-tissue or multi-organ events. Cell-intrinsic and cell-extrinsic timer mechanisms both contribute to the overall timing of events during development, and we have described the utility of experimental embryology (in particular, heterochronic grafting) in distinguishing between these modes of developmental timer control.

Through changes in morphology and structure throughout ontogeny, tissues are brought into and out of close apposition, allowing for the controlled timing of developmental signalling events. Signalling events provide an important level of control for developmental timing, being converged on by both low-level events, including gene expression, as well as dramatic changes in the morphology of the embryo. We propose that tissue tectonics is a key mechanism that integrates timing information across scales of organization within the embryo during development. As we have seen, changes to the timing of a signalling event can have a dramatic effect on morphology; for example, in the development of the emu wing, in the development of the cavefish eyefield and in the development of the dolphin flipper. It is conceivable that the diversity of gastrulation stage embryonic forms in vertebrates is associated with changes to the timing of developmental events, as tissue tectonics will be markedly different [71].

In summary, developmental timing is a highly distributed phenomenon in developmental biology that is coordinated over diverse levels of organization. A huge number of timer mechanisms, some intrinsic and others extrinsic, work together to reproducibly time developmental events in the embryos of a given species. We have shown the importance of inter-cell signalling events as a point of control and coordination across these levels of organization and have argued for the importance of tissue tectonics (the movement of signalling and responding tissues relative to one another) in timing events.

## Data accessibility

This article has no additional data.
